# A genome on shaky ground: exploring the impact of mitochondrial DNA integrity on Parkinson’s disease by highlighting the use of cybrid models

**DOI:** 10.1007/s00018-022-04304-3

**Published:** 2022-05-05

**Authors:** Martin Lang, Anne Grünewald, Peter P. Pramstaller, Andrew A. Hicks, Irene Pichler

**Affiliations:** 1 Institute for Biomedicine, Eurac Research, Affiliated Institute of the University of Lübeck, Bolzano, Italy; 2 Luxembourg Centre for Systems Biomedicine, University of Luxembourg, L-4362 Esch-sur-Alzette, Luxembourg; 3 Department of Neurology, University Medical Center Schleswig-Holstein, Campus Lübeck, Lübeck, Germany

**Keywords:** Parkinson’s disease, Mitochondria, Mitochondrial genome, mtDNA, Cybrids

## Abstract

Mitochondria play important roles in the regulation of key cellular processes, including energy metabolism, oxidative stress response, and signaling towards cell death or survival, and are distinguished by carrying their own genome (mtDNA). Mitochondrial dysfunction has emerged as a prominent cellular mechanism involved in neurodegeneration, including Parkinson’s disease (PD), a neurodegenerative movement disorder, characterized by progressive loss of dopaminergic neurons and the occurrence of proteinaceous Lewy body inclusions. The contribution of mtDNA variants to PD pathogenesis has long been debated and is still not clearly answered. Cytoplasmic hybrid (cybrid) cell models provided evidence for a contribution of mtDNA variants to the PD phenotype. However, conclusive evidence of mtDNA mutations as genetic cause of PD is still lacking. Several models have shown a role of somatic, rather than inherited mtDNA variants in the impairment of mitochondrial function and neurodegeneration. Accordingly, several nuclear genes driving inherited forms of PD are linked to mtDNA quality control mechanisms, and idiopathic as well as familial PD tissues present increased mtDNA damage. In this review, we highlight the use of cybrids in this PD research field and summarize various aspects of how and to what extent mtDNA variants may contribute to the etiology of PD.

## Introduction

Mitochondria are cellular organelles that carry their own genome, the mitochondrial DNA (mtDNA), and their own protein synthesis machinery [1, 2]. These organelles, often labelled as the ‘power stations’ of the cell, play an important role in the regulation of a plethora of key cellular processes, including energy metabolism, calcium homeostasis, oxidative stress response, and cellular signaling towards cell survival or death. Neurons have special energetic needs to fuel axonal and dendritic transport, the release and re-uptake of neurotransmitters, and vesicle trafficking at synapses. Therefore, it does not come unexpectedly that mitochondrial dysfunction has emerged as one of the most prominent cellular processes involved in neuronal dysfunction and neurodegeneration in Parkinson’s disease (PD) [3].

PD is the most common neurodegenerative movement disorder, with a particularly high and rising prevalence in Europe and North America [4, 5]. The neuropathological hallmarks of PD include the progressive loss of dopaminergic neurons in the *substantia nigra pars compacta* (*SNpc*) and the occurrence of proteinaceous inclusions known as Lewy bodies (LBs) in the remaining neurons [6, 7]. The subsequent dopamine deficiency in the basal ganglia triggers cellular and synaptic dysfunction, leading to the classical parkinsonian motor symptoms, which include tremor, rigidity, bradykinesia, and postural instability. In addition, significant non-motor symptoms, such as mental health issues, sleep disorders, pain, or fatigue are associated with PD and can largely precede the motor symptoms [5]. At present, only symptomatic treatments are available, with levodopa (L-Dopa) as the gold standard, and so during treatment, the disease pathology continues to progress.

PD has been widely accepted as a multifactorial disorder, with both genetic and environmental factors playing an important role, and age being the biggest risk factor [8]. The large majority of PD cases is classified as idiopathic (iPD, i.e., with an unknown etiology, ~ 90%), and about 10% of cases represent rare monogenic forms with Mendelian inheritance patterns, with more than 20 genes identified to date [9]. However, several of these genes lack replication and are, therefore, still debated. In addition, about 90 common variants, which exert an increase in heritable PD risk estimated between 16 and 36%, were identified by genome-wide association studies [10]. Several environmental risk factors, including pesticide exposure and traumatic brain injury, are also linked to the risk of developing PD. Conversely, cigarette smoking, caffeine intake and physical activity have been described as protective factors [11]. Interestingly, a recent study has shown that tobacco use is associated also with later age at onset (AAO) in patients carrying the p.G2019S mutation in *LRRK2*, and smoking intensity and duration was also correlated with AAO [12].

The earliest report linking mitochondria and PD dates back to the 1980s, when two incidents of drug abuse were reported, where patients showed typical signs of L-Dopa-responsive parkinsonism after injecting synthetic heroin contaminated with MPTP (1-methyl-4-phenyl-1,2,3,6-tetrahydropyridine) [13, 14]. As a highly lipophilic compound, MPTP can pass the blood brain barrier and, once in the brain, it is converted into 1-methyl-4-phenylpyridinium (MPP^+^), which selectively enters dopaminergic neurons through the dopamine transporter [15, 16]. Within neurons, MPP^+^ concentrates in mitochondria, where it inhibits complex I of the respiratory chain (NADH:ubiquinone oxidoreductase), leading to reactive oxygen species (ROS) production and ATP depletion [17, 18]. The link between mitochondrial malfunction and PD was further corroborated some years later when reduced activities of complexes I, II and IV were found in *postmortem SNpc* homogenates of iPD patients [19–21]. Complex I is dysfunctional also in platelets, lymphocytes and fibroblasts of PD patients [22–24]. Moreover, evidence is mounting that exposure to environmental inhibitors of the electron transport chain, like the pesticide rotenone, is associated to an increased risk for PD [25, 26], which further links mitochondria with PD. Ultimately, genetic evidence also implicates mitochondrial function as an integral disease component, since several familial PD genes are associated either directly or indirectly with mitochondrial function [27]. Mutations in *PINK1*, *PRKN* (Parkin), *DJ-1*, and *CHCHD2* directly impact mitochondrial function, whereas mutations in *SNCA* (α-synuclein), *LRRK2*, *ATP13A2*, *FBXO7*, *VPS13C, VPS35,* and *GBA1* genes mostly show indirect effects on mitochondrial function (reviewed recently in [28]). Interestingly, mutations in the mitochondrial Ubiquinol-Cytochrome C Reductase Core Protein 1 (*UQCRC1*) gene have been found recently to cause autosomal dominant parkinsonism with polyneuropathy, characterized molecularly by mitochondrial complex III dysfunction [29]. However, mutations in this gene were not replicated in other PD cohorts [30–32].

The human mitochondrial genome (mtDNA) According to the endosymbiont hypothesis, mitochondria and their organellar DNA have **evolved** from an alpha-proteobacterial ancestor through intracellular symbiosis with an essentially eukaryotic host cell. During evolution, most genetic material from the endosymbiont was transferred to the nucleus, while a small, compact genome is retained. Several possible reasons for the retention of mitochondrial DNA (mtDNA) have been put forward, including the possibility for localized and dynamic control of the central energetic machinery, retention of genes with high GC content and genes encoding hydrophobic proteins that otherwise may be difficult to properly import and assemble into protein complexes [33].

The **human mtDNA** is a small, circular 16,569 bp DNA molecule encoding 13 protein subunits of the mitochondrial respiratory chain complexes, including 7 complex I, 1 complex III, 3 complex IV, and 2 complex V subunits that are essential to the assembly and function of the respiratory chain [1, 34]. In addition, it encodes 2 ribosomal RNAs (rRNA) and 22 transfer RNAs (tRNA). A ~ 1.1 kb noncoding region (D-Loop) regulates replication and transcription of the mitochondrial genome through the action of nuclear-encoded proteins, such as TFAM (Transcription Factor A, Mitochondrial).

Mitochondrial RNAs are **transcribed** within the mitochondria from both mtDNA strands as polycistronic precursor transcripts and processed mostly through the “punctuation model”, whereby tRNAs, that are interspersed between protein-coding genes, are excised to release individual RNAs, which undergo further maturation events [35]. Transcriptomics data show a wide variation in gene expression of single mtDNA-encoded genes, even though they are derived from the same polycistronic transcript, suggesting sophisticated posttranscriptional regulatory mechanisms [36]. In general, mitochondrial **gene expression** varies widely between cell types, tissues, and individuals, following the energy demand of a cell and is coordinated with expression of nuclear genes encoding mitochondrial proteins [36–38]. Abundant small noncoding RNAs are present in mitochondria and probably play additional regulatory functions in the coordination of mitochondrial gene expression [36].

Furthermore, **epigenetic regulation** of mtDNA provides an intriguing possibility to metabolically regulate mtDNA at the site of oxidative metabolism. However, mtDNA epigenetics is a highly debated topic. Even though there is evidence for the presence in mitochondria of DNA methyltransferases and methylated cytosine residues [39–44], mtDNA methylation levels were generally reported to be low and likely not involving CpG sites [39, 45, 46]. However, mitochondrial metabolism can influence nuclear DNA methylation, which, in return, can affect mitochondrial function (reviewed in [44]).

While the mtDNA is not organized around classical histones, it is uniformly coated by TFAM and other DNA-binding proteins involved in mtDNA regulation [47] to form the mitochondrial **nucleoid**. Most mammalian mitochondrial nucleoids contain a single copy of mtDNA, are localized to the inner mitochondrial membrane, and their distribution is coordinated with mitochondrial fission and fusion events [48].

Cells carry multiple copies of mtDNA ranging between ~ 100 and 100,000, depending on the cell type [49, 50]. Due to the high copy number, genetic sequence variations can either be present in all molecules within a cell or tissue (homoplasmy), or reference and variant alleles can coexist at varying ratios, a condition known as **heteroplasmy**. The fraction of mutant alleles (heteroplasmy level) will affect the pathological consequence of an mtDNA mutation. mtDNA genetics further distinguishes itself from nuclear genetics by **uniparental inheritance**. In mammals, mtDNA is normally inherited exclusively from the mother, allowing for genealogical studies to trace the maternal lineage. Recently, some evidence suggested biparental inheritance of mtDNA in rare cases [51], which was, however, challenged by additional studies [52, 53]. mtDNA sequence variations can be passed on as germline variants or they can be acquired as somatic changes throughout the life cycle of an individual.

## Cybrid cell models

The question of how and to what extent mtDNA variants contribute to the pathology in PD has long been debated and is still not clearly answered. Genetic manipulations of the mtDNA are difficult to achieve, which complicates direct molecular studies and the creation of model systems. A sophisticated model to study the contribution of mitochondrially encoded genes to cellular phenotypes is represented by cytoplasmic hybrids (cybrids). After their first description in 1989 by King and Attardi [54], cybrid cells became important models to study mtDNA contributions to pathologies, such as mitochondrial disorders, cancer, and neurodegenerative diseases.

**Fig. 1:**
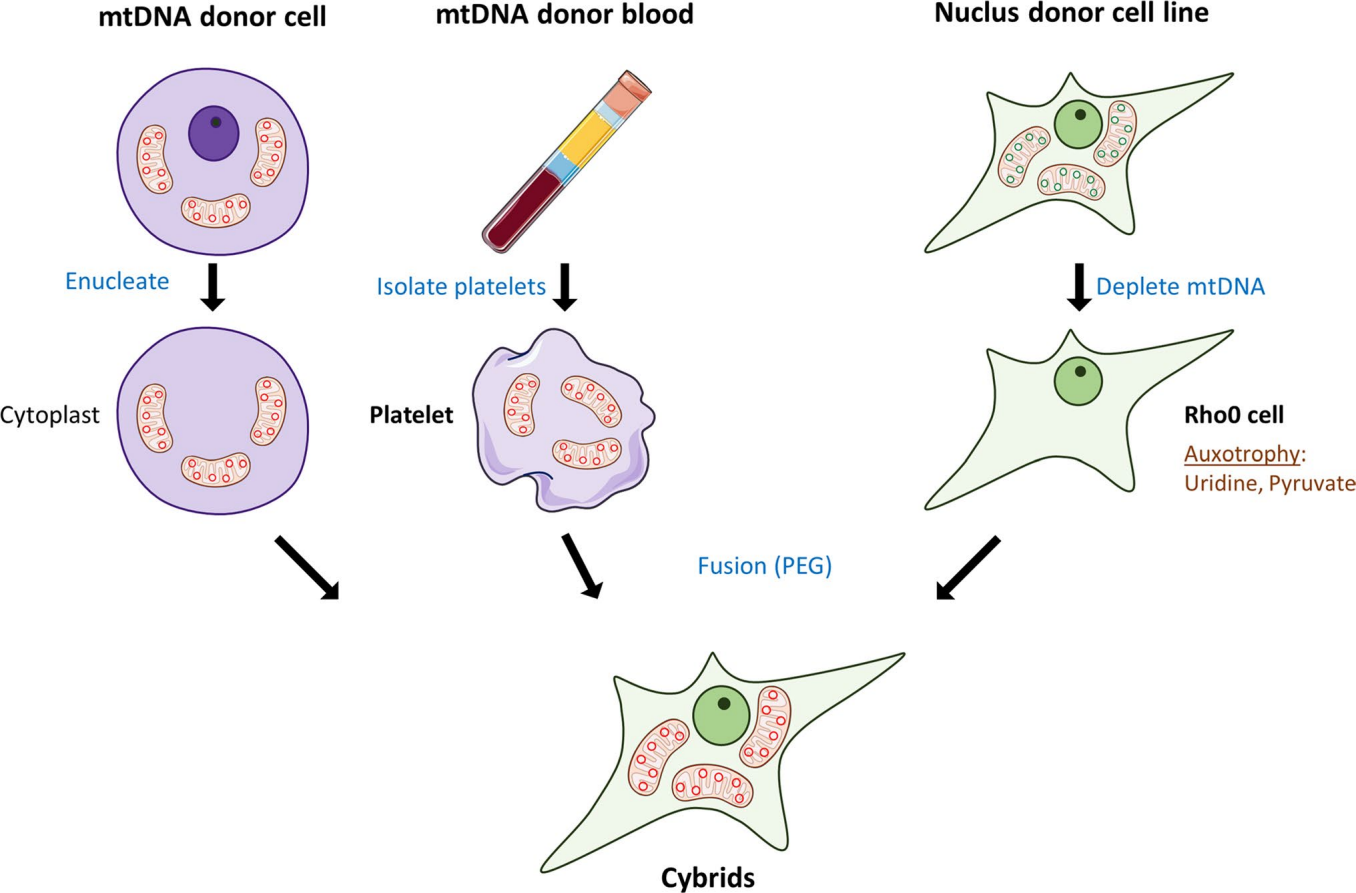
Schematic outline of cybrid cell model generation. The creation of cybrid cells involves the collection of cells devoid of nuclear DNA either by enucleation of the mitochondria donor cells (cytoplasts) or by the collection of platelets from blood. Cells providing the nuclear genome for the resulting cybrids are obtained by ridding cells of mtDNA through chemical (e.g., Ethidium Bromide) or genetic (e.g., mitochondria-targeted restriction enzymes or base excision repair pathway enzymes) means. Selection of resulting mtDNA-depleted Rho0 cells can be performed based on their auxotrophy for uridine and pyruvate. Finally, cybrid cells are created by polyethylene glycol (PEG)-mediated cytoplasmic fusion of mitochondria-donor cytoplasts or platelets with nuclear-donor Rho0 cells and positively selected against uridine and pyruvate auxotrophy

The creation of cybrid cells involves the elimination of mtDNA from recipient cells, which is mostly achieved by long-term treatment with Ethidium Bromide or through enzymatic methods, such as mitochondrially targeted restriction enzymes [55] or base excision repair pathway enzymes [56]. Cells lacking mtDNA are called Rho0 (ρ0) cells and are characterized by respiratory chain deficiency. Rho0 cells, although missing the mitochondrial genome, still contain mitochondrial organelles that can carry out several biochemical functions. The creation of cybrid cells is then achieved by repopulating Rho0 cells with donor mitochondria through cytoplasmic fusion with cytoplasts. Donor mitochondria can be obtained from platelets or from enucleated cytoplasts derived from cells carrying the mitochondria of interest. Studies of patient-derived mitochondria are mostly carried out by fusion of Rho0 cells with platelets that naturally lack the nucleus but contain mitochondria and mtDNA. Because Rho0 cells lack a functional respiratory chain, they are auxotrophic for pyruvate and uridine, a property that can be exploited to negatively select for Rho0 cells or positively select for cybrids repopulated by donor mitochondria. In this context, uridine is thought to supplement de novo pyrimidine synthesis, blocked at the dihydroorotate dehydrogenase step in Rho0 cells, while pyruvate may establish a favorable cellular redox balance [54]. Figure 1 gives an overview of the cybrid model generation process.

## Cybrid studies of mitochondrial physiology in PD

Cybrid models were successfully used to prove the influence of mtDNA variants on cell function for many inherited mitochondrial disorders (reviewed in [57]). The recognition of mitochondrial defects, and in particular complex I deficiency in PD, led to the speculation that the mitochondrial genome may contribute to disease etiology. Therefore, cybrids were early on and extensively employed to shed light on several aspects of PD disease biology, such as mitochondrial defects within PD patient cells, genetic alterations in PD, signaling pathways and therapeutic options. While initial cybrid models were generated using osteosarcoma cell lines [54], the first neuronal cybrid models were established in 1996 by creation of respiratory-deficient SH-SY5Y neuroblastoma cell lines and repopulation with exogenous human platelet mitochondria. Such cells could successfully be differentiated into cells with neuron-like phenotypes [58]. These experiments were rapidly followed by repopulation of neuronal Rho0 cells (human neuroblastoma SH-SY5Y and teratocarcinoma NT2 cells) with mitochondria from iPD patients and demonstrated that patient-derived mitochondria are responsible for reduced respiratory chain complex I activity, increased ROS, lack of antioxidant enzyme upregulation, abnormal morphological mitochondrial ultrastructure, alterations in calcium homeostasis and increased susceptibility to cell death under stress [59–62]. Yet, in-depth and comprehensive genetic analyses of the mtDNA in these models were not reported. More recent biochemical analyses of NT2 cell cybrids showed that basal oxygen consumption was similar in PD and control cybrids, but maximal respiratory capacity was decreased, and proton leakage was increased in PD cybrids [63]. Differentiation of SH-SY5Y cybrids into neuronal cells allowed for controlled genetic conditions and led to the observation that PD cybrid neuronal cells had a lower differentiating potential compared to controls and were characterized by reduced axonal transport of mitochondria and higher dopamine release [64, 65].

However, the application of cybrid technology to a larger number of iPD patients evidenced that such models showed large heterogeneity between clones from patient and control subjects. While several mitochondrial physiological parameters including respiratory chain defects were mostly confirmed in cybrids carrying PD patient-derived mitochondria, substantial variations between clones were observed in histochemical and functional assays. Such assays included respiratory chain function, as measured by oxygen consumption and respiratory chain enzyme activities, genomic properties, as determined by mtDNA copy number assays and a non-sequence-specific heteroplasmy assay (i.e., Surveyor Nuclease®), as well as physiological properties, including mitochondrial movement and depolarization [66–70]. This variability may underlie the fact that some studies could observe only little effect of PD mitochondria on cellular phenotypes or no significant alterations of electron transport chain subunit protein levels [67, 69]. A comprehensive summary of cybrid studies evaluating the role of mtDNA alterations on mitochondrial functions in PD was recently reviewed by Buneeva et al. [71]. Importantly, studies aiming to evaluate physiological changes based on subtle somatic mtDNA alterations warrant cautious interpretation of results and should carefully control the experimental design with age-matched controls, since many studies have shown that aging alone leads to the accumulation of somatic mtDNA mutations in various tissues [72, 73].

## Cybrid studies of molecular defects in PD

Nevertheless, cybrid models have proved to be valid and very useful tools to investigate signaling pathways and disease cascades involved in PD etiology. Indeed, a comparison between iPD cybrid cells with brain tissue samples from iPD patients showed a strong correlation between protein expression profiles of nuclear and mtDNA-encoded electron transport chain genes, suggesting that cybrid cells reliably mimic bioenergetic properties of the PD brain [74]. Pathophysiological investigations showed that cybrids with PD patient-derived mitochondria have an increased propensity to undergo **apoptosis** and have an altered response to cellular stress [59, 75]. Studies of PD cybrids also corroborated the importance of protein aggregates in the pathology of PD. Long-term cultures of cybrid cell lines can develop fibrillar and vesicular **inclusions** with a typical protein composition of PD-related Lewy bodies [76]. While the expression of the yeast single-subunit NADH dehydrogenase (Ndi1) in SH-SY5Y cybrids increased mitochondrial respiration and expression of mitochondrial genes, it was not sufficient to rescue cybrid Lewy body formation. Also, mitochondrial function remained related to the mtDNA and not the Lewy body status of selected cell clones [70]. Furthermore, cybrid models have allowed to link mitochondrial function with **intracellular trafficking** and associated homeostasis processes, such as autophagy. Disruption of microtubule-dependent intracellular trafficking in PD cybrids can impair autophagic degradation, as well as mitochondrial dynamics and health [77, 78]. Conversely, ATP depletion in PD cybrids can disrupt microtubule protein motors, leading to axonal transport disruption and microtubule depolymerization, which can trigger α-synuclein oligomerization [79].

## Cybrid studies of inherited mtDNA variants in PD

The possible **causal contribution of inherited mtDNA variants** to PD was exemplified in a few cases and followed up in cybrid studies. Swerdlow et al. provided early evidence for a potential contribution of mtDNA variants to the PD phenotype in a family with maternally inherited parkinsonism [80]. The comparison of several cybrid lines established with mitochondria from maternal and paternal descendants of the family showed a lower complex I activity, increased ROS levels and abnormal mitochondrial morphology in cybrids derived from maternal descendants. The potential influence of pathogenic mtDNA variants on parkinsonism was further illustrated by a young patient with a complex phenotype including features of parkinsonism. This patient carried high levels of an *MT-CYB* frameshift mutation, which was responsible for respiratory, metabolic, and biochemical deficiencies in cybrid cells [81]. Also another inherited heteroplasmic mtDNA variant, m.T1095C, affecting *MT-RNR1* was described in a family with maternally inherited parkinsonism, sensorineural deafness, and neuropathy [82]. Cybrids with mitochondria from an affected member of this family demonstrated depletion of mitochondrial glutathione and decreases in complex II/III enzymatic activity, in addition to increased apoptosis under aminoglycoside antibiotic [83].

**Fig. 2:**
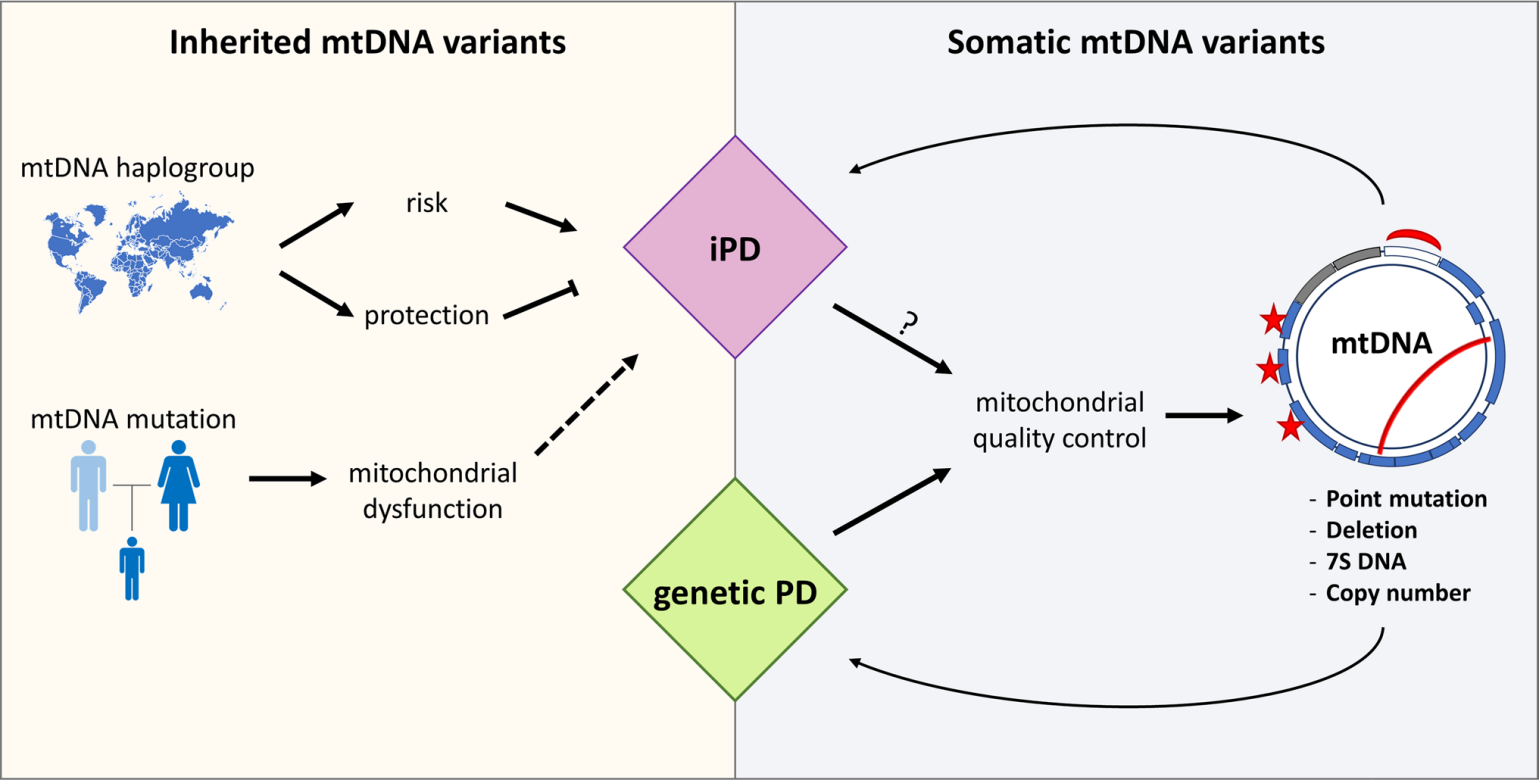
Outline of the contribution of inherited and somatic mtDNA variants to idiopathic and genetic forms of PD. mtDNA genome alterations can contribute to PD as inherited or somatic variants. 1) Inherited mtDNA variants are maternally transmitted and can influence mitochondrial function. While mtDNA haplogroup-driving variants can increase the risk or protect against PD, pathogenic mtDNA mutations decrease mitochondrial functions and can lead to PD-related phenotypes in patients. 2) Somatic mtDNA variants can accumulate in post-mitotic tissues, such as the brain. Various types of mtDNA variants, including point mutations and base damage, mtDNA deletions, transcription/replication‐associated 7S DNA molecules, and variations in mtDNA copy numbers have been investigated in PD patient tissues. While no clear genotype–phenotype correlation has been established to date, somatic mtDNA variations accumulate in affected tissues, such as neurons of iPD patients, suggesting an underlying defective mitochondrial quality control mechanism. In genetic forms of PD, nuclear gene mutations can directly affect mitophagy pathways (e.g. *PRKN*, *PINK1*). This can exacerbate the accumulation of somatic mtDNA variants, which may add to the mitochondrial dysfunction phenotype and aggravate the pathogenic cascade in both genetic and idiopathic forms of PD

Cybrid cell lines carrying known **pathogenic mtDNA mutations**, such as those found in patients with mtDNA-derived mitochondrial disorders, have been used to study cellular reactions to mitochondrial insults. For instance, by investigating cybrid cells carrying mtDNA mutations leading to respiratory chain complex I or complex IV defects, it was observed that aggregated α-synuclein can induce mitochondrial dysfunction specifically through inhibiting complex I [84]. Conversely, the overexpression of wild-type Parkin, known to affect mitochondrial quality control, can ameliorate certain mitochondrial parameters in long-term cybrid cultures of cells carrying heteroplasmic mtDNA mutations (e.g., *COXI* mutation [85]). Physiologically, the Parkin–mitophagy pathway seems to be upregulated in cells carrying pathogenic mtDNA mutations, such as the MERRF (Myoclonic Epilepsy with Ragged-Red Fibers) syndrome-causing m.A8344G mutation, perhaps as a protective feedback mechanism [86] (Fig. 2).

More in general, some **population genetic variants** in the mtDNA have been shown to contribute to the genetic component of complex disorders, including PD. For example, cybrid studies found that cells carrying mitochondria from haplogroup H displayed differences in several mitochondrial physiology parameters (mtDNA and mtRNA levels, mitochondrial protein synthesis, cytochrome oxidase activity and amount, oxygen consumption, mitochondrial inner membrane potential and growth capacity) when compared with those of the haplogroup Uk [87]. This concept underlines the fact that mtDNA evolution is an active process and not all variants are detrimental. Clinical investigations and cybrid experiments provided data supporting the role of variant mtDNA in protecting against PD. For example, haplogroup B5 with the m.A10398G (*MT-ND3* p.T114A) and m.G8584A (*MT-ATP6* p.A20T) variants was proposed to protect against PD, and cybrids with these variants were more resistant to rotenone and had higher thresholds to induce autophagy and apoptosis, compared to cybrids carrying mitochondria from B4 haplogroup without these variants [88] (Fig. 2).

In addition to strictly investigating the contribution of mtDNA variants to PD-related phenotypes, cybrid cells were also used to explore mitochondrial dysfunction in familial PD. A distinct mitochondrial phenotype could be observed between cybrids carrying mtDNA from iPD patients as compared to cybrids from patients with a familial autosomal dominant form of PD, caused by *SNCA* mutations. Cybrids created from PD patients from a Contursi kindred in Southern Italy with an *SNCA* mutation did not manifest complex I deficiencies, when compared to iPD cybrids. However, in line with most PD-related cellular models, mitochondrial oxidative stress was still observed [89].

Taken together, while mtDNA sequence variants may influence PD pathogenesis, mtDNA mutations as ultimate genetic cause of PD have not been established to date.

## Alternatives to cybrid models

Due to technical challenges and the heterogeneous nature of cybrid models, in recent years, they were not extensively applied in PD research. New methodological advances of genome editing strategies, such as the clustered regularly interspaced short palindromic repeats (CRISPR)/Cas9 system, have allowed the development of alternative models that can be adapted to specific research questions. To investigate genetic contributions to the phenotype of known pathogenic PD-driving nuclear gene mutations or PD disease-relevant genes, isogenic cell models can be developed by precise genome editing. Restoration of wild-type alleles in PD patient-derived cells or the insertion of PD-relevant mutations in control cells allow to explore genetic contributions to disease etiology [90]. Meanwhile, genetic engineering of mutations in nuclear-encoded mitochondrial genes can help to examine mitochondrial contributions to the PD phenotypes [91]. In addition, to study the phenotypic contribution of mtDNA variants, big progress has been made over the last years in developing tools for precise genome editing of the mtDNA. Such genome editing technologies include restriction endonucleases, zinc finger nuclease, transcription activator-like effector nucleases (TALEN), including the Double-stranded DNA Deaminase toxin A- derived cytosine base editor (DdCBE), the CRISPR system, and prokaryotic Argonaute proteins (pAgos)-based systems (reviewed in [92]). While such mtDNA genome editing strategies still need to be improved, models combining PD-relevant nuclear gene alterations with engineered mtDNA mutations may further contribute to the dissection of mitochondrial contributions in PD pathogenesis.

## mtDNA alterations in Parkinson’s disease

### mtDNA alterations in iPD

While several of the genes causing monogenic PD can be shown to directly affect mitochondrial quality, nuclear genome mutations can explain only a small fraction of PD. Alterations of the mtDNA are, therefore, an appealing target to investigate as a contributing factor for PD development. Once acquired, somatic mutations of the mtDNA are expected to accumulate in specific tissues due to clonal expansion or as a consequence of continued replication during life. Such mutations might be more likely to significantly expand in post-mitotic tissues, such as the brain, as compared to peripheral tissues, like blood cells, which are characterized by a quick turnover and potentially by a selection against cells with mitochondrial dysfunction caused by mutated mtDNA [93–95]. In general, a threshold of 60–90% heteroplasmic/mutant mtDNA results in phenotypic expression of mitochondrial dysfunction; however, this threshold depends on the type of mutation as well as cell and tissue types [96, 97]. In post-mitotic neurons, a lower level of heteroplasmy might be sufficient to induce phenotypic effects in patients.

In iPD patients, four different types of mtDNA alterations, namely mtDNA point mutations, mtDNA deletions, variations in mtDNA copy number and transcription/replication‐associated 7S DNA molecules have been investigated in *postmortem* brains or peripheral tissues including blood and skeletal muscle [98–104], but no specific rare variant and no clear genotype–phenotype correlation have been found so far that would establish a direct relationship between germline mtDNA variants and PD (Table1). However, a high proportion of multiple heteroplasmic mtDNA deletions were found to accumulate in the *SNpc* and other brain regions with age and were significantly more abundant in iPD patients [105, 106]. Higher levels of somatic mtDNA deletions were also found in another study investigating dopaminergic *SN* neurons of individuals with iPD as compared to age-matched controls [107]. Furthermore, ultra-deep sequencing revealed a large and heterogeneous pool of low-frequency mtDNA deletions in addition to a few (1–4) abundant species of mtDNA deletions in single dopaminergic neurons of two PD patients [108]. Moreover, in single *SN* neurons of iPD patients, a reduction of 7S DNA molecules, which bind to the D-Loop region during mtDNA transcription and replication, and mtDNA depletion were observed, which was more pronounced in neurons with severe complex I deficiency. On the contrary, in fibroblasts of iPD patients, accumulation of 7S DNA, low mtDNA replication, high heavy strand transcription and low mtDNA release was detected [109]. Using a histochemical approach able to detect abasic sites in the mtDNA, in which there is a loss of either a purine or a pyrimidine base, *postmortem* tissue sections of iPD patients showed a selective accumulation of mtDNA damage in nigral dopaminergic neurons [110] (Table1, Fig. 2). Of note, when investigating single cells of three different brain regions (putamen, frontal cortex, *SN*) of Alzheimer’s disease patients and controls, mtDNA deletion load was higher in *SN* neurons compared to the other brain regions, possibly pointing to a specific susceptibility of dopaminergic neurons to accumulate mtDNA damage, which was independent of the disease state [111]. Interestingly, the same selectivity of mtDNA damage in midbrain neurons was detected in rats exposed to the pesticide rotenone, where the mtDNA lesions appeared before any signs of neurodegeneration under conditions of mitochondrial impairment [110]. Such mitochondrial phenotypes and genotypes correlated with a decrease in mtDNA transcription factors TFAM and TFB2M [112]. Previous studies have shown that heterozygous loss of *Tfam* in mouse cells and tissues lead to a reduction in mtDNA copy number, respiratory chain deficiency, and increased oxidative mtDNA damage [113, 114].

**Table 1:** Summary of main mtDNA alterations described in PD, their common detection methods, and related molecular consequences

Genetic alteration	Description	Detection methods	Genotype in PD	Consequence
mtDNA copy number	Number of mtDNA molecules per cell	Southern blot; rtPCR; droplet digital PCR	Mostly decreased in PD-related tissues	Linked to respiratory chain deficiency
7S DNA quantity	Transcription/replication‐associated third DNA strand at D-Loop region	Southern blot; rtPCR; droplet digital PCR	Reduction or accumulation of 7S DNA in PD	Alteration of replication/transcription switch of mtDNA
Major arc deletion	Large mtDNA deletion, including the "common deletion" (ΔmtDNA4977), involving *ATP8*, *ATP6*, *COXIII*, *ND3*, *ND4L*, *ND4*, and *ND5* genes	Southern blot; rtPCR	Increased fraction of mtDNAs with deletion in PD-related tissues	Altered respiratory chain activity
Point mutations	Single nucleotide variants and small indels	Direct sequencing (Sanger); deep sequencing (NGS); non-sequence-specific heteroplasmy assay (e.g., Surveyor Nuclease®), histochemistry (abasic nucleotides)	Mostly increased mtDNA damage and accumulation of mtDNA variants in PD-related tissues	Multiple effects: common polymorphic variants to pathogenic effects on respiratory chain proteins

Some studies have shown that mtDNA integrity and copy number specifically in *SN* neurons is correlated also with complex I and cytochrome c oxidase (COX) deficiency, suggesting that mtDNA alterations might be linked to impaired mitochondrial respiration [105, 112]. The particular susceptibility of dopaminergic neurons to mitochondrial dysfunction may be due to multiple factors, including their morphology, such as large axonal arbors and their unmyelinated or only lightly myelinated axons, their high overall pace-making function, and the energy-demanding and oxidatively challenging dopamine-producing metabolism (reviewed in [115]). Increased energy requirements of *SN* neurons in PD might be impacted by additional mitochondrial stressors, like mtDNA impairments, possibly contributing to the unique selective vulnerability, although more data on mtDNA integrity in *SN* neurons need to be collected. Compared to nuclear DNA, mtDNA is more vulnerable to ROS because it is physically close to ROS-producing sites, such as respiratory chain complexes I and III, and it lacks a protective chromatin structure. In addition, mitochondria possess fewer DNA repair pathways, and the mitochondrial DNA polymerase (POLG) shows different properties at incorporating modified nucleotides than nucleus DNA polymerases (reviewed in [116]). Accordingly, after exposing cultured cells to oxidative stress, the damage to mtDNA was higher and persisted longer than that to nuclear DNA, indicating that mtDNA is a cellular target for ROS [117]. This pathway may lead to a vicious cycle whereby damaged mitochondria increase ROS production, which exacerbates mtDNA damage. This mechanism was exemplified in a model of acute ischemic kidney injury, where mitochondrial ROS reduced the abundance of TFAM, resulting in reduced mtDNA synthesis and mitochondrial biogenesis, leading to decreased energy metabolism [118].

On top of known PD risk genes, nuclear genetic variants may contribute to mitochondrial defects and mtDNA alterations described in PD. A polygenic enrichment of rare nonsynonymous variants in genes belonging to pathways controlling mtDNA maintenance has been shown to influence the risk of iPD [119]. Similarly, the analysis of mitochondria-specific polygenic risk scores evidenced a cumulative small effect of common genetic variation within genes implicated in mitochondrial function on PD risk and AAO [120].

### mtDNA alterations in genetic forms of parkinsonism

mtDNA alterations may also be the driving force of the mitochondrial phenotype in the presence of nuclear gene mutations involved in parkinsonism. Mutations in *POLG*, which lead to the accumulation of multiple mtDNA deletions over time, have been associated with Alpers-Huttenlocher syndrome (AHS) in children and chronic progressive external ophthalmoplegia (CPEO) in adults [121–124]. Parkinsonism occurs in a fraction of patients of both disorders. In addition to mtDNA deletions, *POLG* variants were also correlated with decreased mtDNA copy numbers in blood cells of PD patients [104]. Notably, neuronal cell loss was observed in patients with mutations in *POLG*, but not in other mitochondrial disorders, suggesting that patients with acquired (somatic) mtDNA mutations are more susceptible to neuronal degeneration compared to individuals with inherited mtDNA mutations [125].

It is becoming clear that PINK1 and Parkin are involved in several aspects for mitochondrial quality control and mtDNA maintenance (see BOX: Mitophagy). 7S DNA levels were found to be reduced in induced pluripotent stem cells (iPSC)-derived neuronal progenitor cells of patients with *PRKN* mutations as compared to age-matched controls, while the abundance of major arc deletions was unchanged in these individuals [126]. By genome-wide chromatin immunoprecipitation experiments, Parkin was found to interact with the mtDNA, most probably through an indirect interaction with the mitochondrial transcription factor TFAM [127]. Furthermore, in cell lines stably overexpressing Parkin, it was shown to increase mtDNA replication and transcription of mitochondrial genes [127, 128]. Parkin, together with PINK1, also controls mitochondrial biogenesis through Parkin interacting substrate (PARIS) and downstream regulation of Peroxisome proliferator-activated receptor gamma coactivator 1-alpha (PGC-1α) in a mechanism that proved to be critical for the survival of dopaminergic neurons [129–131].

Furthermore, also neural cells derived from iPSCs from patients carrying a homozygous or a heterozygous *LRRK2* p.G2019S mutation, or asymptomatic individuals carrying a heterozygous *LRKK2* p.R1441C mutation, showed significantly increased mitochondrial lesions as compared to control cells. After zinc finger nuclease-mediated repair of the *LRRK2* p.G2019S mutation in iPSCs, mtDNA damage was not detected anymore in differentiated neural and neuroprogenitor cells, demonstrating that *LRKK2* mutations are linked to mtDNA lesions in PD patients and asymptomatic mutation carriers [110, 132]. Recently, when investigating mtDNA integrity in fibroblasts of manifesting and non-manifesting carriers of the *LRRK2* p.G2019S mutation, higher levels of somatic major arc deletions were detected in manifesting mutation carriers [133]. In addition, mtDNA copy number was found to be elevated in fibroblasts of manifesting mutation carriers as compared to non-manifesting carriers, and 7S DNA levels were reduced in *LRRK2* p.G2019S mutation carriers independent of disease status [103]. These data suggest the involvement of mtDNA dyshomeostasis in the penetrance of *LRRK2* mutations. Extending on these findings, alterations in the mtDNA maintenance pathway, including mtDNA copy number and 7S DNA, were found in *PRKN*-mutant iPSC-derived neurons, highlighting these mtDNA markers as potential modifiers of disease manifestation [134]. Furthermore, in *LRRK2* p.G2019S mutation carriers, cell-free mtDNA concentration in cerebrospinal fluid correlated with α-synuclein levels, which was higher in mutation carriers compared to iPD patients [135] (Table1, Fig. 2).

In addition, PD-associated α-synuclein mutations let to mitochondrial DNA damage, altered mitochondrial transport and morphology, and reduced mitochondrial membrane potential [136–138]. Metabolic and cellular bioenergetics impairments were linked to accumulation of pathogenic α-synuclein aggregation at the mitochondrial membranes [139, 140] and a potential functional link was provided by an α-synuclein overexpressing mouse model that presented increased mtDNA deletions and oxidative DNA damage, which was related to the mitochondrial protein transport machinery [141]. mtDNA variation might, therefore, be a risk factor for increased phenotype severity and/or earlier AAO in genetic forms of parkinsonism.

Mitophagy Owing to the central function of mitochondria within the network of cellular metabolic processes, well conserved mechanisms have evolved to recognize and remove dysfunctional mitochondria and to integrate these mechanisms with cellular homeostasis pathways. Besides regulation of mitochondrial biogenesis, fission and fusion, as well as turnover of single mitochondrial proteins and protein complexes, bulk disposal of mitochondria by the macro-autophagy machinery—known as mitophagy—is part of the mitochondrial quality control process.

The mitophagy pathway has been elucidated through the study of the PD-associated genes *PINK1* and *PRKN*. While knockout mouse models for these genes do not show evident neuronal phenotypes, *Drosophila* models carrying *Pink1* and *Parkin* knockouts were successfully used to show that Pink1 and Parkin work in the same pathway to control mitochondrial health [142–145]. Based on this evidence, it was established that the PINK1 kinase accumulates on the outside of damaged mitochondria [146–149]. Under normal conditions, the PINK1 precursor protein is recruited to mitochondria for internalization through the outer mitochondrial membrane TOM complex [150] to the inner mitochondrial membrane TIM complex. At the inner mitochondrial membrane, PINK1 is readily cleaved by PARL protease to generate an internal phenylalanine [151], which triggers its rapid degradation by the proteasome [152]. Under conditions of mitochondrial depolarization, PINK1 cannot be cleaved by PARL, which leads to its accumulation at the outer mitochondrial membrane. There, PINK1 phosphorylates Parkin in its ubiquitin-like domain at Ser65, recruiting Parkin to the mitochondria and activating its ubiquitination activity towards other mitochondrial proteins [153–155]. Mitochondria marked by ubiquitinated groups are recognized by LC3 cargo receptors (e.g., NDP52, Optineurin, Tax1BP1, NBR1 and p62) that direct tagged dysfunctional mitochondria through LC3 binding towards phagophores for degradation by the autophagy machinery [156].

Mitophagy has mostly been studied in cell culture models under artificial mitochondrial membrane depolarizing conditions. However, it can also be triggered by metabolic stress in a complex interplay between mitochondrial biogenesis and degradation pathways. Mitochondrial network remodeling during development also involves mitophagy, as shown for several cell types (erythrocytes, cardiomyocytes, oocytes) in various organisms (mammals, flies, worms) (reviewed in [157]). Recent works in vitro and in vivo have shown the occurrence of basal mitophagy under physiological conditions, which is often PINK1-Parkin independent [158–161]. Parkin-independent mitophagy involves other E3 ubiquitin ligases, such as Gp87, SMURF1, SIAH1, MUL1, and RIH1. These mechanisms largely follow the downstream pathway described for the PINK1–Parkin pathway, whereby mitochondrial proteins are tagged for degradation, autophagy adaptors (e.g. UKL1) are being recruited to expand autophagosomal membranes, and cargo recognition proteins such as LC3 are being anchored to autophagosomes, thereby triggering lysosomal degradation (reviewed in [157].

## Impact of impaired mitophagy on mtDNA dynamics in PD

As exemplified by the fact that genetic forms of PD can be driven by mutations in central mitophagy genes (e.g. *PRKN*, *PINK1*), mitophagy defects resulting in a decline of mitochondrial function have emerged as common feature in PD (reviewed recently by [162]). In general, a decline of mitochondrial fitness is observed during aging, which may be driven by ROS or a drop in the removal of malfunctioning mitochondria. Evidence for this stems from research that showed the accumulation of abnormal mitochondria and a rise in ROS production upon knockout of autophagy genes in immune cell models [163]. Likewise, accumulation of mtDNA double strand breaks were shown to underly mitochondrial dysfunction in mouse cardiomyocytes, in which *Mitofusin2* knockout was used to model mitophagy deficiency [164]. On the reverse, increasing mitophagy can increase the selection against defective mitochondria, as evidenced by long-term overexpression of Parkin that decreased the heteroplasmy load of a deleterious COXI mtDNA mutation in cybrid cells [85].

As outlined above, there is evidence that somatic rather than inherited mtDNA mutations might be involved in the pathogenesis of PD and that Parkin and PINK1 might contribute to the removal of mtDNA mutations through mitophagy [165]. However, the exact mechanism of the interplay between these factors is poorly understood.

Mitophagy defects have been connected also to the pathogenesis of various inflammatory diseases, including neuroinflammation, an early event in the pathogenesis cascade of PD (reviewed in [166]). A dysfunctional mitochondrial quality control pathway, as caused by *PINK1* or *PRKN* mutations, has recently been proposed to be linked to increased risk for PD through inflammation. The intra- and extracellular release of damage-associated molecular patterns (DAMPs), including mtDNA fragments, from damaged mitochondria is a potent trigger of the innate immune system. mtDNA can trigger inflammation through intracellular sensors, including nucleotide-binding oligomerization domain (NOD)-like receptor (NLR), Toll-like receptor (TLR), and the cytosolic cyclic GMP-AMP synthase (cGAS)-stimulator of interferon genes (STING) DNA sensing pathway. Inhibition of mitophagy has been shown to cause accumulation of mitochondrial ROS and damaged mitochondria, which in turn can trigger inflammation through the activation of the MAPK pathway [167] or the NLRP3 inflammasome [168]. Activation of these cellular response pathways can trigger an interferon response, followed by secretion of pro-inflammatory cytokines (e.g. IL-6), a condition recently described in PD patients carrying *PRKN/PINK1* mutations, and therefore provides a rationale for the connection between mitochondrial dysfunction and inflammation in PD [165, 169].

## mtDNA homeostasis in in vivo models of PD

Translational studies of human diseases, including PD, benefit greatly from in vivo animal models. Chemically induced rodent models of PD rely mostly on toxins that affect either mitochondrial function (e.g., MPTP) or dopamine metabolism (e.g., 6-hydroxydopamine, 6-OHDA). Similarly, genetic mouse models have focused on engineering genes known to cause familial PD or to cause mitochondrial dysfunctions.

The MitoPark mice, with disruption of the gene for mitochondrial transcription factor A (TFAM) in dopaminergic neurons, are characterized by a marked depletion of mtDNA, impairment of oxidative phosphorylation, dopaminergic neuron degeneration and motor deficits that mimic human parkinsonism [170]. Similarly, the “TwinkPark” mice, characterized by knockout of *Prkn*, together with *SN*-specific mtDNA deletions due to a mutation in the mtDNA helicase *Twinkle* (*C10orf2*), presented decreased mitochondrial function in *SN* cells, accompanied by increased neurobehavioral deficits and parkinsonian phenotypes compared to single mutant control animals [171]. Notably, mutations in *Twinkle* were described in association with CPEO/parkinsonism patients [172, 173].

In the Mutator mouse model, which possesses a proofreading-deficient form of POLG, resulting in time-dependent accumulation of multiple deletions in the mtDNA, neuroprotective compensatory mechanisms were observed at the mitochondrial level. These data suggested that somatic mtDNA deletions per se are not sufficient to trigger *SNpc* dopaminergic neuron death in animal models [174]. In contrast, in the mtDNA mutator *Drosophila* model that showed mitochondrial dysfunction, shortened lifespan, progressive locomotor deficit, and loss of dopaminergic neurons, a positive selection of deleterious mtDNA mutations was observed [175]. After crossing the Mutator mouse with a *Prkn* knockout mouse, which both by themselves do not show neurodegeneration, mitochondrial dysfunction and PD pathology became apparent. This was linked to a greater predicted pathogenicity score of mtDNA mutations. Surprisingly, however, the overall abundance and type of these mutations (synonymous vs. nonsynonymous) was unchanged [176]. This study highlights a key role of mtDNA mutations in dopaminergic neurons and a protective role of Parkin, since its loss synergizes with mitochondrial dysfunction resulting in neurodegeneration. The *Prkn* knockout:Mutator mouse model was also used to show a role for mitophagy at mitigating inflammation through the STING pathway [165].

Attempts at modeling PD in vivo based on mitochondrial dysfunction through genetic disruption of nuclear-encoded complex I subunits evidenced a complicated picture of complex I dysfunction in PD. *Ndufs4* knockout mouse models with a mild complex I deficiency in vivo displayed an altered dopamine metabolism but did not present neurodegeneration phenotypes [177, 178]. On the contrary, a recently described model with *Ndufs2* knockout in dopaminergic neurons (MCI-Park mice) showed that complete complex I disruption can lead to an axonal-first progressive neurodegeneration that produces a human-like parkinsonism in which the loss of nigral dopamine release makes a critical contribution to motor dysfunction. This model allowed the authors to follow the PD disease cascade over time and between different brain regions [179].

PINK1 and Parkin have been shown to play important roles in the mitochondrial quality control through the mitophagy pathway and may, therefore, also be involved in the selection against deleterious mtDNA mutations. However, their role in vivo under physiological conditions is still debated. Comparisons of heteroplasmic mtDNA variants between wild type, *Pink1* knockout and *Parkin* knockout mice showed an increase in early onset single nucleotide variants in *Pink1* knockout mice and an increased variant allele frequency of mtDNA mutations in *SN* of aged *Pink1* and *Prkn* knockout mice [180]. Furthermore, in a transgene-based *Drosophila* model carrying a heteroplasmic mtDNA deletion in adult muscle tissue, genetic induction of the Pink1-Parkin pathway resulted in a selective decrease in mtDNA deletion molecules that was at least partially dependent on autophagy [181]. These studies indicated that selection against dysfunctional mitochondria can be achieved in vivo in post-mitotic tissues. Another elegant model to study the effect of mtDNA variants and their selection process in vivo was provided by *Drosophila* carrying a heteroplasmic, temperature-sensitive COXI mutation induced by a mitochondria-targeted restriction enzyme and cytoplasm transfer [182, 183]. While Parkin was not involved in the selection process of deleterious mtDNA in this model, PINK1 did preferentially accumulate on mitochondria enriched for the pathogenic mtDNA mutations and was required for selection of healthy mitochondria. However, rather than inducing the canonical mitophagy pathway, PINK1 was shown to inhibit local protein translation through phosphorylation of Larp, which induced preferential replication of normal mitochondria and hampered the propagation of defective organelles [184, 185]. Collectively, these data provide evidence for an interplay between PD-related nuclear gene functions and mtDNA alterations in the etiology of PD.

## Treatment strategies in PD aimed at mitochondrial dysfunction and mtDNA

In recent years, the microbiome–gut–brain axis has gained particular attention as underlying pathological mechanism in PD, linked through metabolic alterations, bacterial metabolites, and inflammatory responses (reviewed in [186]). In this context, **pro- and prebiotics** may represent promising options to prevent or ameliorate PD symptoms through modulation of inflammatory pathways or metabolic functions [186]. The use of dietary supplements to improve mitochondrial function has long been applied to primary mitochondrial disorders and has been suggested also for PD. A functional interaction between diet and mtDNA has been proposed in fly models, where mtDNA plays a role in modulating diet-dependent fitness [187]. Also in humans, a higher diet quality was associated with a greater whole blood-derived mtDNA copy number [188].

In the context of PD, dietary supplement research focused primarily on the antioxidant pathway as potential therapeutic approach to counteract the detrimental effects of ROS and to preserve mitochondrial function (reviewed in [189]). For example, **coenzyme Q10** (ubiquinone) that functions as an electron carrier in the electron transport chain shows potent antioxidant properties, and hence an effect also on mtDNA content and integrity [190]. While several preclinical studies showed beneficial effects of coenzyme Q10 dietary supplementation in PD models, meta-analyses of randomized, placebo-controlled trials in human PD found no evidence that coenzyme Q10 improved motor-related symptoms or delayed the progression of the disease when compared to placebo [191, 192]. Similarly, a mitochondrially targeted quinone, **MitoQ** (mitoquinone), proved beneficial in several preclinical models, but not in clinical trials in PD patients [193]. Furthermore, **polyphenols** have been proposed as beneficial molecules against PD due to their antioxidant and anti-inflammatory actions, but conclusive pharmacological benefits are outstanding (reviewed in [194]). A more direct effect on cellular metabolism and influence on mtDNA variations can be expected by metabolic fuels, such as **proteins and carbohydrates**. In fly models, it was suggested that a diet with a low protein to carbohydrate ratio and rich in certain fatty acids would be beneficial for cells with respiratory chain complex I deficiencies and Parkin mutations [195, 196]. Also, **calorie restriction** may play a role in mtDNA integrity and prolonging lifespan through the regulation of multiple molecular pathways, with direct implications for NDDs, including PD (reviewed in [197]). However, the effect of dietary supplements on mitochondrial metabolism and mtDNA are mainly based on preclinical models. Translation of such treatment strategies to clinical practice have mostly failed to show significant benefits and will warrant better patient stratification and improved biomarkers.

In addition to dietary adjustments, **exercise** has been shown in preclinical and clinical studies to provide a health benefit to PD patients, which can at least in part be explained by mitochondrial metabolic improvements and may potentially involve the mitochondrial genome. Notably, in the MitoPark mouse, which is characterized by inactivation of TFAM, exercise was shown to improve behavioral parameters and nigrostriatal dopamine input as well as oxidative phosphorylation [198]. Furthermore, exercise prevented alterations of mitochondrial biogenesis signaling (PGC-1α/NRF-1/TFAM) and respiratory chain activity in a PD rat model [199]. However, exercise-induced motor improvements in PD were associated with metabolic changes but not with muscle mtDNA content [200].

## Summary

There is accumulating evidence demonstrating that mitochondrial dysfunction either directly underlies the neurodegenerative disease process in PD or constitutes one part of a complex series of events. The establishment of human cybrid cell lines provided the opportunity to show direct molecular consequences of the expression of mtDNA variants from iPD patients in vitro. Although no rare inherited mtDNA mutation was linked to PD as yet, studies performed in *postmortem* brains or peripheral tissues including blood and skeletal muscle identified four different types of somatic mtDNA alterations, namely mtDNA point mutations, mtDNA deletions, variations in mtDNA copy number and transcription/replication‐associated 7S DNA molecules. Also, in different tissues of genetic PD patients, higher levels of somatic mtDNA mutations were detected. Genetic mouse models suggest that dysfunctional degradation of mitochondria carrying mtDNA mutations through the process of mitophagy might be associated with the accumulation of somatic mutations at different heteroplasmy levels in different tissues. A schematic overview of contributions of inherited vs. somatic mtDNA variations to the pathogenesis of PD is represented in Table 1 and Fig. 2.

Methodological advances are adding to our understanding of biological and genetic basis of PD. For example, recently established long-read sequencing methodologies (i.e., Nanopore sequencing) allowed for the investigation of mtDNA methylation architecture and provided some evidence for CpG methylation differences between tissues and between PD patients and healthy controls [45]. Application of refined genomic and biochemical methods will be needed to accurately measure mtDNA base modifications and adducts in order to quantify stress-induced mtDNA alterations and better understand mtDNA repair mechanisms. As such, deep sequencing of tissues and cell line models, ideally by long read sequencing technologies to capture structural variants, will help to clarify the picture of mtDNA alterations in PD. Furthermore, transcriptomics and sequencing studies at the single cell level should provide information on the somatic mitochondrial genetic architecture of brain tissues. Performance of such studies in appropriate animal models, where early stages of the disease can be evaluated may allow to place mitochondrial and mtDNA dysfunction into the timeline of the disease cascade of PD.

Mitochondrial and mtDNA alterations result in metabolic changes of the affected cells. To better connect genetic and physiological alterations in PD, but also to properly select and stratify patients for clinical trials, it will be important to pair genetic analyses with studies of metabolic alterations in model systems and brain tissues. Functional imaging techniques that allow the assessment of mitochondrial metabolism and bioenergetic disturbances may proof helpful tools and can be performed longitudinally to compare different stages of disease (e.g., reviewed in [189]). The altered mitochondrial metabolism may also offer a therapeutic handle against PD, and comprehensive metabolomics characterizations of PD models are providing the bases for such approaches [201]. Treatment options could be inspired by recent work in the field of mitochondrial disorders, where it was shown that metabolic restrictions can suppress pathological mtDNA replication. By limiting the energy output of glycolysis with a glucose analog such as 2-Deoxy-D-glucose (2DG) or 5-thioglucose, wild-type mtDNA was selected over mutant mtDNA copies in the context of a pathogenic mtDNA mutation [202]. Furthermore, accelerating the removal of damaged mitochondria by pharmacologically enhancing mitophagy may provide an additional therapeutic approach against PD [162].

Mitochondrial function is also tightly connected with a proper function of the immune system and mitochondrial dysfunctions can trigger inflammation, e.g., through release of mtDNA fragments. The understanding that inflammation plays an important and early role in PD pathogenesis suggests that targeting the immune system with anti-inflammatory drugs may provide another promising therapeutic avenue against PD [165].

An intact mitochondrial genome is central to proper mitochondrial function, but mtDNA is vulnerable to insults. Mitochondrial impairment appears to be centrally involved in the neurodegenerative process of PD, independent of the root cause of the disease in a single patient. Our understanding of the underlying genetic and pathophysiological mechanism is, therefore, providing the basis for therapeutic approaches against PD.

## Data Availability

Enquiries about data availability should be directed to the authors.
